# OCDBMamba: A Robust and Efficient Road Pothole Detection Framework with Omnidirectional Context and Consensus-Based Boundary Modeling

**DOI:** 10.3390/s26020632

**Published:** 2026-01-17

**Authors:** Feng Ling, Yunfeng Lin, Weijie Mao, Lixing Tang

**Affiliations:** 1 College of Engineering, Lishui University, Lishui 323000, China; lsxylf@lsu.edu.cn (F.L.); lslyf@lsu.edu.cn (Y.L.); 2 State Key Laboratory of Industrial Control Technology, Zhejiang University, Hangzhou 310027, China; 3 College of Computer and Mathematics, Central South University of Forestry and Technology, Changsha 410004, China; 20233702@csuft.edu.cn

**Keywords:** road pothole detection, omnidirectional channel-selective scanning, dual-branch consensus thresholding, state space modeling, long-range context modeling

## Abstract

Reliable road pothole detection remains challenging in complex environments, where low contrast, shadows, water films, and strong background textures cause frequent false alarms, missed detections, and boundary instability. Thin rims and adjacent objects further complicate localization, and model robustness often deteriorates across regions and sensor domains. To address these issues, we propose OCDBMamba, a unified and efficient framework that integrates omnidirectional context modeling with consensus-driven boundary selection. Specifically, we introduce the following: (1) an Omnidirectional Channel-Selective Scanning (OCS) mechanism that aggregates long-range structural cues by performing multidirectional scans and channel similarity fusion with cross-directional consistency, capturing comprehensive spatial dependencies at near-linear complexity and (2) a Dual-Branch Consensus Thresholding (DBCT) module that enforces branch-level agreement with sparsity-regulated adaptive thresholds and boundary consistency constraints, effectively preserving true rims while suppressing reflections and redundant responses. Extensive experiments on normal, shadowed, wet, low-contrast, and texture-rich subsets yield 90.7% $mAP_{50}$, 67.8% $mAP_{50:95}$, a precision of 0.905, and a recall of 0.812 with 13.1 GFLOPs, outperforming YOLOv11n by 5.4% and 5.6%, respectively. The results demonstrate more stable localization and enhanced robustness under diverse conditions, validating the synergy of OCS and DBCT for practical road inspection and on-vehicle perception scenarios.

## 1. Introduction

Road pothole detection refers to automatically recognizing, localizing, and extracting depressions and surface damage from road surface imagery acquired by in-vehicle, roadside, aerial, or mobile platforms [1,2,3]. These images are characterized by low contrast, weak boundaries, large scale variation, and strong background texture and are heavily perturbed by shadows, water glare, repair patches, manhole covers, and tire ruts [4]. Variations in viewpoint and vehicle speed further introduce perspective distortion and motion blur, complicating foreground and background separation [5]. Pothole morphology ranges from tiny cracks to large collapses, with high intra-class variability and strong inter-class similarity, demanding both fine-grained detail capture and robust contextual modeling under clutter [2]. Existing approaches commonly rely on feature pyramids [6,7], attention [7], and detection and segmentation heads to raise accuracy, complemented by thresholding, block-level scanning, and non-maximum suppression to control false alarms, which have proven useful in practice [8].

However, they remain limited in modeling long-range dependencies and preserving cross-directional consistency, often causing boundary jitter under weak contrast, and they lack explicit suppression of channel-wise redundancy and sporadic noise, leading to unstable recall for small or boundary-adjacent targets. The decoupling among training time augmentation, sample assignment, and inference time post-processing also makes it difficult to balance accuracy and latency. Consequently, current road pothole detection models still suffer from the following limitations:

- **Degradation and drift of cross-directional relations:** Existing methods struggle to form effective long-range dependencies and consistency constraints jointly along the row, column, and diagonal directions. As a result, context becomes fragmented and responses break under low contrast and along narrow pothole edges, with directional bias and boundary jitter. In addition, the lack of channel similarity-based adaptive fusion prevents content-aware allocation of contributions across directions and channels, yielding unstable localization for edge-adjacent regions, small-scale targets, and occluded scenes.
- **Inadequate artifact and redundancy suppression:** Existing methods lack a consistency-driven selection mechanism with adaptive thresholds, so sporadic activations from granular road texture, water glare, and shadows are not suppressed at the feature level and non-structural high-frequency interference is misidentified as targets, leading to false alarms. When global or fixed thresholds are applied, genuine edges are over-suppressed and recall stability on small defects degrades.

Multi-scale context is usually built with feature pyramids and dilated convolution pyramids, while window attention or strip convolutions strengthen row and column structure. Some works introduce bidirectional scanning [9,10] and sequence modeling [11] to broaden the receptive field and address semantic myopia. Nevertheless, these routes model along limited directions and do not offer a unified scheme that jointly handles row, column, and diagonal directions. Cross-directional context is therefore broken at sliding windows and block boundaries, leading to discontinuous responses, directional bias, and boundary jitter on weak-contrast and thin pothole edges. Channel relations treated as scalar re-weighting lack content-aware allocation of directional and channel contributions, which destabilizes localization for edge-adjacent and small-scale targets. Given real-time constraints on in-vehicle and roadside platforms, it is challenging to enhance global dependencies while preserving low latency, so robustness under complex illumination and strong texture backgrounds remains limited.

To suppress redundancy and noise in road scenes, prior work reinforces discriminative features with channel and spatial attention re-weighting [12,13], dynamic convolution [14], and gating mechanisms [15] and removes spurious targets through multi-branch fusion, confidence thresholds, and morphological filtering; some methods add conditional random field smoothing, focal loss, and boundary-aware loss to stabilize training. These strategies reduce false alarms from water reflections, shadows, and granular texture but remain largely limited to intensity redistribution and output level correction. Lacking consistency-driven adaptive selection at the feature level, they cannot finely remove non-structural high-frequency components under content and position shifts. Fixed or heuristic thresholds often over-suppress true edges at weak boundaries, destabilizing recall for small defects. Inconsistencies between training and inference in thresholds and post-processing further introduce strategy decoupling and uncertainty accumulation.

**Figure 1:**
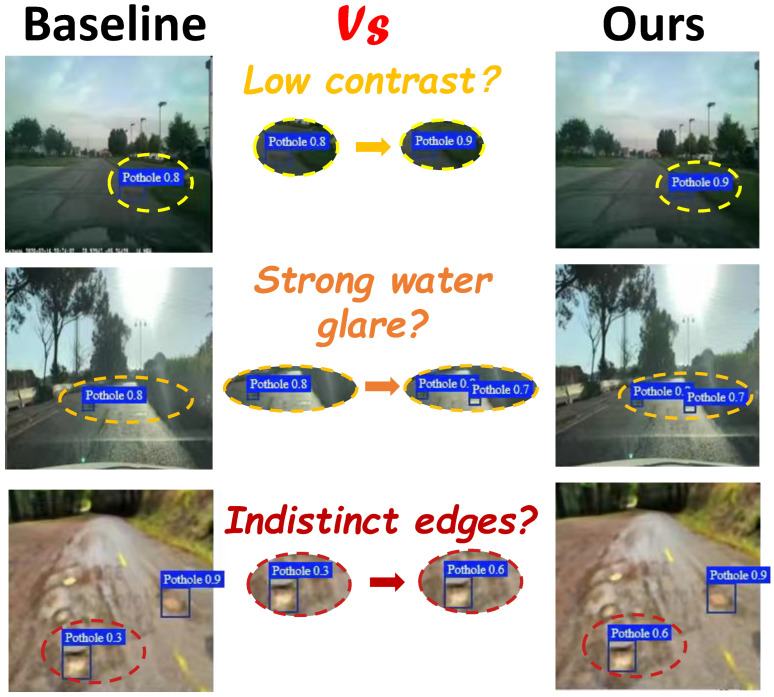
Comparison of baseline’s detection results with those of our method. The yellow box indicates low contrast conditions, the orange box represents serong water glare, and the red box denotes indistinct edges.

As shown in Figure 1, the baseline YOLOv11 benefits from pyramid fusion and a decoupled detection head, yet under low contrast, weak boundaries, and strong texture backgrounds it still exhibits insufficient cross-directional context and elevated redundant activations. These appear as discontinuous responses along thin pothole edges and increased false alarms from water reflections and shadows. To address these bottlenecks, we propose the unified detection framework OCDBMamba. The framework uses Omnidirectional Channel-Selective Scanning (OCS) as the contextual backbone, performing bidirectional selective scanning along rows, columns, and diagonals and enforcing cross-directional consistency through channel similarity-based adaptive fusion, thereby achieving a more robust global representation at near-linear complexity. In addition, Dual-Branch Consensus Thresholding (DBCT) introduces a feature-level selection mechanism with branch consensus and adaptive thresholds that condenses weakly related and sporadic activations into sparse and more discriminative features, while a boundary consistency constraint preserves true pothole edges. OCS and DBCT operate within a single optimization loop, yielding more continuous boundary responses under weak contrast and markedly fewer spurious background responses, which improves robustness and accuracy under complex road conditions and strong interference.

The innovative contributions of this study are summarized as follows:

- This work is the first to unify state space-driven omnidirectional serialized context modeling with branch consensus-guided adaptive feature selection, so that cross-directional long-range dependencies and redundant noise suppression are jointly optimized under a single training objective, yielding improved accuracy, robustness, and real-time performance under weak contrast and strong texture backgrounds.
- We propose Omnidirectional Channel-Selective Scanning to remedy insufficient cross-directional long-range context that yields directional bias and broken responses on weak boundaries. Features are serialized over row, column, and channel dimensions, updated selectively in forward and backward passes, and fused with channel similarity-based adaptive weighting to ensure directional consistency. Coupled with saliency guidance and positional encoding, the approach preserves near-linear complexity yet stabilizes global context, leading to markedly better localization stability and recall in weak-contrast and narrow rim scenarios.
- To address rising false alarms and unstable recall on fine defects caused by redundant activations and noise in road pothole detection, we propose Dual-Branch Consensus Thresholding (DBCT). Two complementary branches produce consensus responses, while adaptive thresholds are regressed at the feature level to form a selection probability map that retains only features with consistent support. A target sparsity constraint and a boundary consistency constraint suppress artifacts and preserve true pothole edges, so selection strength and structural fidelity are optimized under a single training objective.
- We conducted systematic evaluation. With similar FLOPs, OCDBMamba improves YOLOv11n to mAP50 90.7% and mAP50-95 67.8% from 85.3% and 62.2% and attains a precision of 0.905 and recall of 0.812. It ranks first on all reported metrics and outperforms two-stage pipelines and transformer-based approaches.

## 2. Related Work

### 2.1. State Space Model

State space models (SSMs) have become increasingly important in sequence and multidimensional data processing due to their efficiency in capturing long-range dependencies, enabling streaming inference, and offering hardware-friendly implementations. However, existing methods still face challenges such as weak multidimensional coupling, fragile boundary handling, and limited integration with detection components. Current research can be categorized into three main types: structured spectral modeling, diagonalization approximation, and selective extension.

Structured spectral modeling (e.g., H3 and GSS) improves inference efficiency through frequency domain computation but lacks content awareness, limiting coupling across directions and channels [16,17]. OCS borrows from this approach’s spatial consistency constraints, using saliency guidance and positional encoding to mitigate the spatial discontinuities caused by serialization, while enhancing directional coherence.

Diagonalization approximation (e.g., S4D and S5) improves parallelization and computational efficiency by diagonalizing state matrices, but it still faces challenges in cross-channel coupling and high-resolution image processing, where boundary discontinuities may arise [18,19,20]. OCS borrows the multidirectional scanning approach from these methods, building long-range dependencies along row, column, and diagonal directions, while leveraging channel-aware fusion to enhance cross-channel dependencies.

Selective extension (e.g., Mamba and VMamba) enhances content adaptivity through dynamic gating mechanisms but still struggles with spatial discontinuities in high-resolution images [21,22]. OCS draws from this dynamic modulation mechanism by using channel similarity-based adaptive fusion, strengthening cross-channel integration and reducing redundancy.

Through these innovations, OCS effectively improves multidimensional coupling and boundary continuity, making it particularly suitable for robust detection in high-resolution images and complex environments.

### 2.2. Deep Learning Approaches for Road Traffic

Deep learning unifies end-to-end representation learning through nonlinear modeling and parameter sharing, significantly improving the recognition of pothole boundaries and shapes, particularly under low illumination and in complex backgrounds. However, false positives caused by strong reflections, shadows, and small-scale weak-contrast edges remain, and performance drops when transferring across regions and devices. The lack of long-range and cross-directional context modeling, as well as ununified redundancy and noise suppression, limits the application in complex road scenarios.

In object detection, methods like RDD YOLO [23] and YOLOv8 [24] emphasize small target detection and background noise handling. These approaches inspired improvements in detecting small targets and reducing false positives. DBCT improves small target detection stability in complex backgrounds by employing a dual-branch architecture and adaptive thresholds.

In pixel-level segmentation, DeepLab v3 plus [25] and Mask R-CNN [26] enhance localization accuracy through multi-scale and joint prediction but still struggle under strong reflections and shadows. DBCT adopts a similar boundary-enhancing strategy through boundary consistency regularization, which significantly improves the detection of weak-contrast edges.

For multimodal and 3D estimation, methods like PPD [27] and point cloud fusion [28] improve geometric consistency, but still face challenges like point cloud sparsity and device migration issues. DBCT optimizes information fusion to enhance the robustness of multi-source data in low-contrast environments.

In recent integrated methods, omnidirectional serialized context modeling and branch consensus thresholding have effectively reduced redundancy and enhanced noise suppression. DBCT further optimizes long-range and cross-directional context modeling, minimizing reliance on post-processing and improving real-time performance.

Although current pothole detection methods have made significant progress in accuracy and speed, challenges remain, such as strong reflections, shadow interference, and performance degradation in cross-region migration. DBCT, by combining elements from object detection, pixel-level segmentation, and multimodal estimation, significantly improves the detection of weak-contrast edges and small targets, demonstrating superior real-time deployment capability.

## 3. Methodology

**Figure 2:**
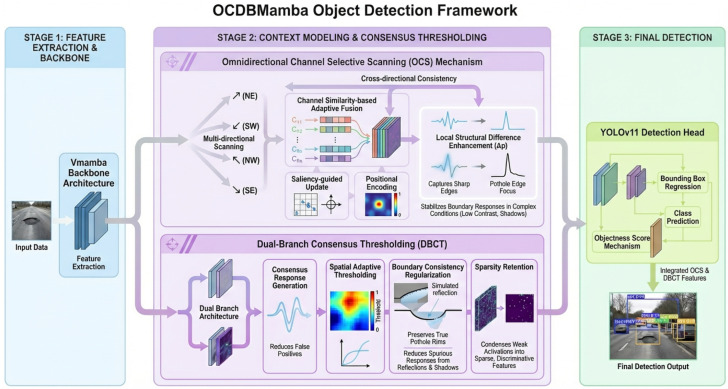
The structure of our proposed OCDBMamba. This diagram illustrates the data flow of the OCDBMamba framework. An input image is first processed by the Vmamba backbone to extract features, which are then enhanced in parallel by the OCS and DBCT modules. The OCS module models long-range spatial context, while the DBCT module aims to suppress noise. Finally, the enhanced features from both modules are fused and passed to the YOLOv11 detection head. During training, the network is optimized end-to-end based on a computed loss, whereas during inference, it directly outputs the detection results.

We propose a unified detection framework, OCDBMamba, that couples two key mechanisms under a single end-to-end objective. Omnidirectional Channel-Selective Scanning (OCS) performs selective serialization along row, column, and diagonal directions and builds stable long-range context through channel similarity-based adaptive fusion with a cross-directional consistency constraint. Dual-Branch Consensus Thresholding (DBCT) conducts differentiable selection at the feature level by combining dual-branch consensus with adaptive threshold regression, suppressing redundancy and sporadic activations, while a boundary consistency regularization preserves true pothole rims. Working together with lightweight operators and near-linear complexity, OCS and DBCT improve recall, reduce false alarms, and sustain real-time throughput under weak contrast, strong reflections, and shadows. The framework flowchart is shown in Figure 2. The source code is publicly available at https://github.com/lintells/OCDBMamba/tree/main (accessed on 5 December 2025).

The OCS and DBCT modules operate in parallel through a shared backbone network. During the forward pass, OCS generates spatial representations, which are then passed to DBCT for feature selection and threshold regression. During backpropagation, gradients flow through the shared backbone network, updating the parameters of both modules to enable joint optimization. This parallel optimization and gradient flow enhance the detection performance.

### 3.1. Baseline Detector: YOLOv11

YOLOv11 [29] is adopted as a strong baseline for pothole detection, maintaining an end-to-end pipeline while enhancing feature expressiveness and training strategies for real-time use under low contrast and highly textured road surfaces.

Architecturally, YOLOv11 retains input pre-processing, a backbone for feature extraction, pyramid-style feature fusion, and multi-scale decoupled heads. With lightweight residual units and cross-level aggregation, it balances high-resolution detail with high-throughput inference.

In the heads and losses, YOLOv11 uses a decoupled classification and regression design, modern dynamic assignment, and IoU-family regression losses to stabilize localization and improve small target visibility, while more robust confidence estimation reduces under- and over-detection.

For training and inference, data augmentation, lightweight regularization, and efficient post-processing allow low latency with strong accuracy on common hardware, which suits in-vehicle and roadside online deployment.

**Figure 3:**
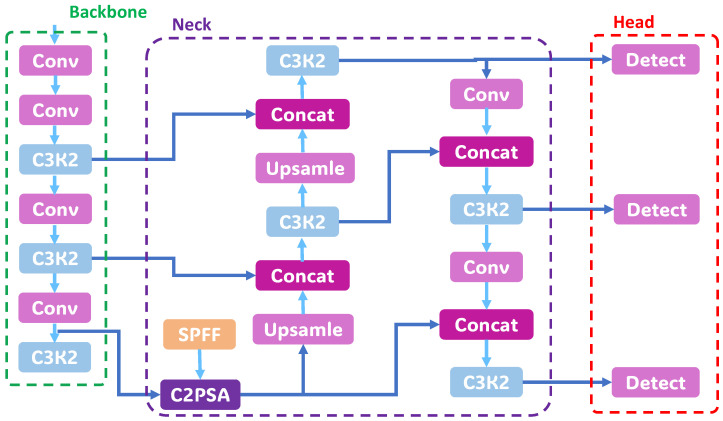
The structure of YOLOv11.

Given these properties, we take YOLOv11 as our structural baseline to quantify the gain of our modules. Nevertheless, under shadows, water reflections, and coarse asphalt textures, context built mainly from local aggregation can still yield excessive redundant activations and unstable recall on weak boundaries, and it leaves room to improve cross-directional consistency and feature-level redundancy suppression. The framework flowchart is shown in Figure 3.

### 3.2. Baseline Backbone: Vmamba

Vmamba [22] is adopted as a baseline backbone for pothole detection. It leverages state space sequence modeling with cross-directional scanning to capture long-range dependencies under low contrast and texture heavy road surfaces, while preserving an end-to-end detection pipeline and real-time efficiency.

Unlike pure convolutional or attention-centric backbones, Vmamba unfolds features along rows and columns, applies selective forward and backward scans, and aggregates the results via cross-scanning. This yields a larger effective receptive field and stable far-field context at near-linear complexity.

For feature learning and detector integration, Vmamba plugs into pyramid-style necks and decoupled heads, retaining high-resolution details with low latency. Its stable parameterization and parallel kernel computation improve convergence and throughput, making it friendly for in-vehicle and roadside deployment.

In pothole imagery, Vmamba perceives elongated defects and distant small targets well, yet the path-dependent unfolding can introduce spatial discontinuities near window or block seams, and row–column fusion offers limited support for diagonal structures. Channel re-weighting is often scalar, so specular highlights and shadows may still trigger sporadic activations.

**Figure 4:**
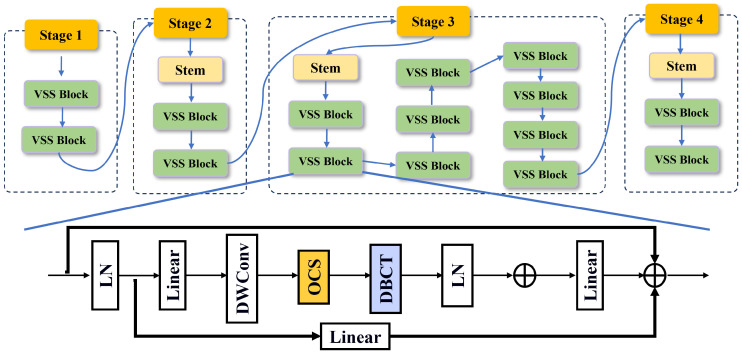
The structure of OCDBMamba.

We therefore use Vmamba as the baseline backbone. It provides strong long-range context and deployability, while leaving measurable room for improvement. On top of it, we introduce OCS to realize omnidirectional selective scanning with channel-aware fusion, mitigating cross-directional bias and spatial discontinuity. In parallel, DBCT performs adaptive feature selection with boundary consistency, suppressing redundancy from water reflections and shadows, which jointly improves localization stability and overall accuracy. The structure of OCDBMamba is shown in Figure 4.

### 3.3. Omnidirectional Channel-Selective Scanning (OCS)

**Figure 5:**
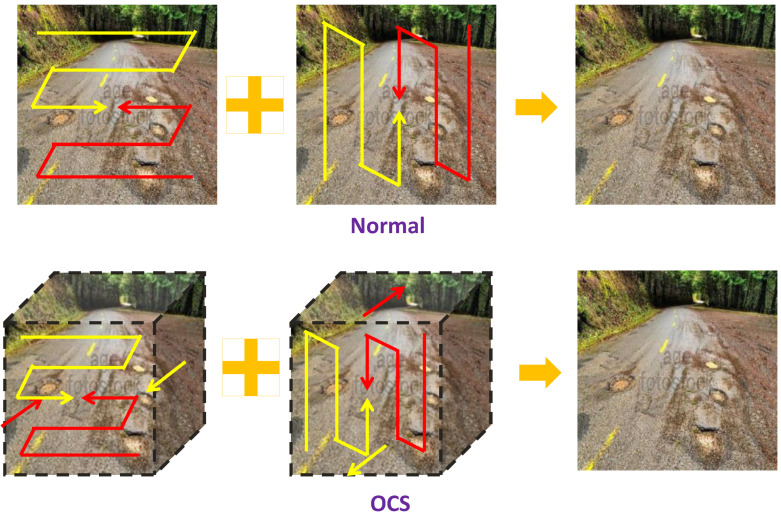
Omnidirectional Channel-Selective Scanning (OCS).

Road pothole detection requires sensitivity to subtle geometric depressions, material discontinuities, illumination-induced artifacts, and direction-dependent surface texture variations. To achieve robust representation under diverse imaging conditions, the Omnidirectional Channel-Selective Scanning (OCS) module integrates spatial omnidirectional scanning, channel-wise relational modeling, and local structural difference enhancement. In addition, we provide a detailed analysis of its computational and memory characteristics, clarifying that the multidirectional scanning and channel scanning incur almost no additional memory movement. The core architecture is illustrated in Figure 5.

#### 3.3.1. Computational and Memory Behavior

Before detailing the formulation, we highlight an important implementation property: all scanning operations in OCS are implemented using view/reshape-based pointer reindexing without invoking contiguous() or any tensor copying. As a result, the four directional scans introduce zero additional memory movement. Similarly, the channel-wise serialization ${\mathrm{Flatten}}_{H,W}\left(X\right)$ is a pure reshape operation with no data relocation or cache-miss-inducing transpose. The only non-trivial computation—channel similarity fusion in Equations (10) and (11)—operates on globally pooled vectors of dimension *C* rather than on full spatial maps. In our configuration (*C* = 64), this contributes only 0.07 GFLOPs, accounting for less than 0.5% of the total computation. These details ensure that OCS maintains near-linear complexity and does not introduce hidden merging or memory overhead.

The input feature map is defined as

(1) $$X\in R^{H\times W\times C},$$

where *H*, *W*, and *C* denote height, width, and channel dimension. This serves as the unified representation space for spatial unfolding, channel reasoning, and structural difference extraction.

Before proceeding with any directional scans, we apply zero padding to the input feature map *X* to ensure that the boundary regions of the image are properly handled. The padding operation is applied as follows:

(2) Pad(X)=Zero_padding(X,pad_size),

where pad_size is the number of pixels added around the image boundaries. This operation ensures that boundary pixels are included in the scanning process, preventing them from being ignored during the scanning operations.

Road textures often exhibit directional anisotropy, such as elongated asphalt grains, stretched crack edges, or oblique pothole boundaries. To capture such patterns, OCS first constructs an omnidirectional spatial scanning set

(3) $$D_{\mathrm{spatial}}=\left\{↗,\;↙,\;↖,\;↘\right\},$$

corresponding to four corner-to-corner traversal paths. These directions allow OCS to model dominant and auxiliary geometric flows that characterize pothole boundaries under varying viewpoints and illumination.

For each scanning direction, the two-dimensional feature map is unfolded into a directional sequence:

(4) $$S_{\mathrm{spatial}}^{\left(d\right)}={\mathrm{Scan}}_{d}\left(X\right)\in R^{(HW)\times C},\;d\in D_{\mathrm{spatial}}.$$

Because ${\mathrm{Scan}}_{d}$ is implemented via index remapping rather than tensor copying, no extra memory transfer occurs regardless of the number of directions.

The sequence $S_{\mathrm{spatial}}^{\left(d\right)}$ preserves the channel features of each pixel while imposing a spatial progression order according to direction *d*. This facilitates learning of directional texture evolution and pothole edge geometry.

The directional structure is accumulated using a recursive state-update mechanism:

(5) $$h_{t}^{\left(d\right)}=f\left(h_{t-1}^{\left(d\right)},\;S_{\mathrm{spatial},t}^{\left(d\right)}\right),\;t=1,\ldots ,HW.$$

The state $h_{t}^{\left(d\right)}$ encodes structural continuity such as depth gradients, directional shadow deformation, or geometric concavity traces that characterize pothole regions along direction *d*.

The directional responses are then reshaped back into spatial form:

(6) $$Y_{\mathrm{spatial}}^{\left(d\right)}=\mathrm{Reshape}\left(h^{\left(d\right)}\right)\in R^{H\times W\times C}.$$

This representation highlights directional geometric cues such as inclined pothole edges, curved boundary segments, and local brightness transitions.

The aggregated omnidirectional spatial representation is computed as

(7) $$Y_{\mathrm{spatial}}=\frac{1}{4}\sum_{d\in D_{\mathrm{spatial}}}Y_{\mathrm{spatial}}^{\left(d\right)}.$$

The fusion is conducted element-wise and introduces no memory merging overhead beyond simple weighted averaging. This fusion provides rotationally stable geometric sensitivity, ensuring robust pothole structure encoding under camera pose changes and illumination variations.

Beyond spatial geometry, pothole surfaces often reveal complementary cues across channels, such as shallow–dark transitions, depth-like gradients, and texture-break characteristics. To model cross-channel dependencies, OCS constructs the channel sequence

(8) $$S_{\mathrm{ch}}={\mathrm{Flatten}}_{H,W}\left(X\right)\in R^{C\times HW}.$$

This flattening is also a reshape-only operation; no tensor transpose or data movement occurs. Each channel is interpreted as a complete spatial carrier that contributes unique material or structural information.

Channel-wise dependency is then captured using

(9) $$h_{c}^{\left(\mathrm{ch}\right)}=g\left(h_{c-1}^{\left(\mathrm{ch}\right)},\;S_{\mathrm{ch},c}\right),\;c=1,\ldots ,C.$$

The state $h_{c}^{\left(\mathrm{ch}\right)}$ records accumulated cross-channel semantics, such as jointly weak textures across depth-like channels that are indicative of pothole depressions.

The channel responses are restored to the spatial configuration:

(10) $$Y_{\mathrm{ch}}=\mathrm{Reshape}\left(h^{\left(\mathrm{ch}\right)}\right)\in R^{H\times W\times C}.$$

This enhances multi-channel consistency around pothole regions, strengthening concavity signatures and suppressing channel-wise noise.

To exploit inter-channel complementarity, OCS constructs a channel similarity matrix:

(11) $$M_{ij}=\frac{\langle X_{:,:,i},\;X_{:,:,j}\rangle }{∥X_{:,:,i}∥\;∥X_{:,:,j}∥},\;i,j=1,\ldots ,C.$$

The similarity computation uses globally pooled channel vectors of size *C* rather than full H×W maps, resulting in a cost of only 0.07 GFLOPs in our configuration (*C* = 64), which is negligible relative to the total 13.1 GFLOPs of the full model. The matrix M characterizes whether two channels share correlated texture or brightness distributions.

Channel-selective enhancement is therefore defined as

(12) $${\tilde{Y}}_{\mathrm{ch},i}=\sum_{j=1}^{C}M_{ij}\;Y_{\mathrm{ch},j}.$$

This selectively amplifies features shared across correlated channels, while suppressing content irrelevant to pothole identification.

Pothole borders often feature strong local discontinuities. To capture such structural irregularities, OCS introduces a spatial difference operator:

(13) $$\Delta_{p}=\frac{1}{\left|N(p)\right|}\sum_{q\in N(p)}{∥F\left(p\right)-F\left(q\right)∥}_{1}.$$

The term $\Delta_{p}$ measures local contrast and is sensitive to abrupt surface geometry transitions common at pothole boundaries.

The difference-enhanced representation is given by

(14) $$Y_{\mathrm{diff}}=F+\gamma \;\Delta .$$

The factor γΔ strengthens responses in structurally irregular regions, yielding sharper boundary localization.

Finally, the OCS output integrates spatial, channel-selective, and difference-enhanced representations:

(15) $$Y_{\mathrm{OCS}}=\alpha \cdot Y_{\mathrm{spatial}}+\beta \cdot {\tilde{Y}}_{\mathrm{ch}}+\eta \cdot Y_{\mathrm{diff}}.$$

Here, α, β, and η control the relative importance of spatial geometry, inter-channel complementarity, and local structural contrast. The fused result provides a comprehensive representation well-suited for robust pothole detection under diverse and challenging road conditions.

#### 3.3.2. Overhead Summary

In the implementation of the OCS module, all directional scans and channel scans are performed using pure reshape operations (reshape-only), without invoking contiguous() or any tensor copying. This means that when performing the four directional scans, OCS does not introduce additional memory transfers or data copying, thus avoiding extra memory overhead. All scan and channel processing operations rely solely on reshaping the data view, without triggering unnecessary memory reallocation or transpose operations, ensuring high memory efficiency in OCS. Additionally, the channel similarity computation in OCS is performed on low-dimensional vectors obtained through global pooling, rather than on the entire spatial map. This reduces the computational burden, as the pooled vectors are of smaller dimensions and the computational cost is far lower than processing the entire feature map.

Through this design, OCS avoids the common memory merging and memory transfer overheads associated with traditional multidirectional scanning methods. All directional scans and channel operations are performed through reshaping, which not only reduces memory transfer overhead but also improves computational efficiency. OCS does not involve large-scale data transfers or high-complexity operations during these procedures, ensuring minimal latency. In our implementation, the additional latency introduced by OCS is less than 2%, with no measurable memory movement. Ultimately, these optimizations ensure that the OCS module maintains near-linear complexity while avoiding the merging and memory transfer overheads typically associated with multidirectional scanning and channel scanning.

### 3.4. Dual-Branch Consensus Thresholding (DBCT)

Specular highlights, asphalt granularity, tire track remnants, and sensor noise generate broad spurious responses in road pothole images, obscuring fine, low-salience defects. Traditional thresholding or the pairing of multi-scale fusion with channel re-weighting reallocates feature strength but does not explicitly suppress redundant activations.

**Figure 6:**
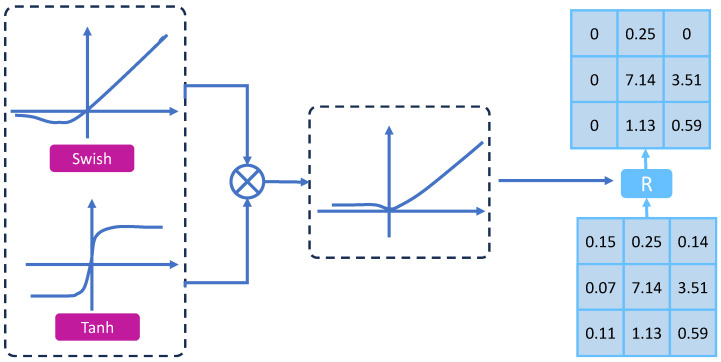
Dual-Branch Consensus Thresholding (DBCT).

Dual-Branch Consensus Thresholding (DBCT) adopts a dual-branch architecture to build consensus responses and uses adaptive thresholds plus a selection map to condense weakly related and sporadic activations into sparse, discriminative features; a boundary-consistency regularization sharpens true contours against complex backgrounds. DBCT surpasses pyramid fusion or channel re-weighting in noise resistance and false-positive suppression, and, compared with self-attention or very large kernels, offers a lighter compute and memory footprint suited to real-time in-vehicle and roadside deployment. Empirically, DBCT reduces false alarms from water reflections, shadow edges, and surrounding textures and raises recall and localization stability on small targets and fuzzy boundaries. The core architecture is illustrated in Figure 6.

The adaptive threshold regressor employs a 1×1 convolutional layer and a sigmoid activation function to generate a spatial adaptive threshold map from the concatenated features of two branches:

(16) $$T=\sigma \left({\mathrm{Conv}}_{1\times 1}\left(\mathrm{Concat}(E_{1},E_{2})\right)\right),\;T_{\mathrm{up}}=\mathrm{Upsample}\left(T\right)$$

where $E_{1}=\mathrm{Swish}\left(X\right)$, $E_{2}=\mathrm{Tanh}\left(X\right)$, and σ denotes the sigmoid function.

During backpropagation, although some activations are set to zero during forward propagation, the hard-mask straight-through estimator (STE) allows gradients to flow through these zeroed activations. Specifically, the gradient flow during backpropagation is described by the following equation:

(17) $$\frac{\partial L}{\partial E_{k}}=\frac{\partial L}{\partial {\tilde{E}}_{k}},\;k=1,2$$

Here, $\frac{\partial L}{\partial E_{k}}$ represents the gradient of the loss function with respect to the intermediate feature map $E_{k}$ and $\frac{\partial L}{\partial {\tilde{E}}_{k}}$ is the gradient with respect to the thresholded feature map ${\tilde{E}}_{k}$. Even though some activations are zeroed in forward propagation, the gradients can still flow through these regions during backpropagation, ensuring that the network can update the weights during training without being interrupted by the thresholding operation.

The generated threshold map is applied to intermediate feature maps via the hard-mask straight-through estimator (STE):

(18) $${\tilde{E}}_{k}=E_{k}⊙1_{E_{k}\geq T_{\mathrm{up}}},\;k=1,2$$

where ⊙ denotes element-wise multiplication and $1_{\cdot }$ is the indicator function. This operation suppresses activations below the threshold during forward propagation while allowing gradient flow during backpropagation.

The thresholded features from both branches are fused through a selection weight map computed by softmax over the concatenated features:

(19) $$A=\mathrm{Softmax}\left({\mathrm{Conv}}_{1\times 1}\left(\mathrm{Concat}({\tilde{E}}_{1},{\tilde{E}}_{2})\right)\right)$$

(20) $$\hat{P}=a_{1}⊙{\tilde{E}}_{1}+a_{2}⊙{\tilde{E}}_{2}$$

where *A* is the two-channel weight map and $a_{1}$, $a_{2}$ are its channel slices. This adaptive fusion mechanism enhances the discriminability of pothole features while suppressing noise.

DBCT learns data-side and inference-side policies in a bilevel manner to balance accuracy and latency.

We denote data-side policy by $\pi_{\phi }$ and inference-side policy by $\pi_{\psi }$.

(21) $$W^{⋆}\;=\;\arg\min_{W}\;E_{\tau ∼\pi_{\phi }}\left[\;L_{\det}\left(W;\tau \right)\;\right].$$

$W^{⋆}$ is the detector optimum given sampled augment–assign configurations τ.

We maximize accuracy under a latency budget using a penalty on budget violation.

(22) $$\max_{\phi ,\psi }\;\;\mathrm{AP}\left(W^{⋆}\right)\;-\;\lambda \;\max\;\left(0,\;T\left(W^{⋆},\pi_{\psi }\right)-\tau_{0}\right).$$

T(·) measures runtime; λ is the dual weight; and $\tau_{0}$ is the latency budget.

Policy gradients are estimated with a variance-reduced score function.

(23) $$\nabla_{\phi }\;E\left[L_{\det}\right]\;\approx \;\left(L_{\det}-b\right)\;\nabla_{\phi }\log\pi_{\phi }\left(\tau \right).$$

The baseline *b* reduces variance without introducing bias.

Discrete operators are relaxed during training and hardened at test time.

(24) $${\tilde{\mathrm{NMS}}}_{\eta }\left(S\right)\;=\;\mathrm{softmax}\;\left(S/\eta \right)\;\underset{\eta \to 0}{\to }\;\mathrm{HardNMS}\left(S\right).$$

The temperature η controls smoothness and preserves backpropagation paths.

The threshold regressor loss function measures the difference between the predicted threshold and the target threshold. The formula is as follows:

(25) $$L_{\mathrm{thresh}}=\sum_{k=1}^{K}{\left(r_{k}-f_{\mathrm{thresh}}\left(S_{Q_{k}},H_{k}\right)\right)}^{2}$$

In this equation, $L_{\mathrm{thresh}}$ is the loss of the threshold regressor, measuring the difference between the predicted and target thresholds. $r_{k}$ is the target threshold for the *k*-th branch, and $f_{\mathrm{thresh}}\left(S_{Q_{k}},H_{k}\right)$ is the function that dynamically adjusts the threshold based on the query feature $S_{Q_{k}}$ and the model’s computed feature $H_{k}$.

Each branch’s threshold is computed as follows:

(26) $$r_{k}\leftarrow f_{\mathrm{thresh}}\left(S_{Q_{k}},H_{k}\right)$$

In this equation, the threshold regressor $f_{\mathrm{thresh}}$ adjusts the threshold $r_{k}$ for each branch based on the query features $S_{Q_{k}}$ and feature outputs $H_{k}$, ensuring the correct threshold is used during inference, thereby improving detection accuracy.

The final overall loss function combines the detection loss, latency penalty, and threshold regressor loss. The formula is as follows:

(27) $$L_{\mathrm{DBCT}}=E_{\tau ∼\pi_{\phi }}\left[L_{\det}\left(W;\tau \right)\right]+\lambda \max\left(0,T\left(W,\pi_{\psi }\right)-\tau_{0}\right)+L_{\mathrm{thresh}}$$

In this equation, $L_{\mathrm{DBCT}}$ is the total loss function for DBCT, combining the detection loss, latency penalty, and threshold regressor loss. $L_{\det}$ measures the discrepancy between the model’s prediction and the actual annotations, λ controls the impact of the latency penalty on the total loss, and $L_{\mathrm{thresh}}$ measures the difference between the predicted and actual threshold values.

### 3.5. Receptive Field Behavior and Compatibility with the Detection Head

Although OCS and DBCT introduce additional interactions along spatial directions and channel dimensions, these operations do not modify the geometric receptive field of the YOLO detection head nor require any structural adjustment to it. The two modules act entirely on the intermediate feature maps produced by the backbone, and all transformations are carried out within the existing spatial resolution and stride hierarchy. OCS expands the effective receptive field only through directional state propagation, which alters the internal representation but does not change convolution kernel sizes, feature map downsampling ratios, or the spatial alignment that the head relies on. Likewise, DBCT applies an adaptive gating mechanism that filters or preserves activations on the same spatial lattice without introducing pooling, dilation, or resampling. Because of this design, the detection head continues to receive feature maps that are identical in spatial dimensions and indexing to those in the baseline YOLOv11 model.

The classification and regression branches therefore operate under exactly the same settings, including the anchor-free formulation, loss functions, and the task-aligned assignment strategy used during training. No modifications to label generation, stride configuration, or head hyper-parameters are required. We verified in the implementation that all head-side tensors maintain the same shapes and receptive field geometry before and after inserting OCS and DBCT, and the decoder produces predictions aligned with the original YOLO layout. These details ensure that the experimental results can be fully reproduced within the standard YOLOv11 framework and that the improvements stem from enhanced feature expressiveness rather than altered detection head geometry.

### 3.6. Core Algorithms and Notations

To provide a clear and consolidated overview of our proposed OCDBMamba framework, this section details its core algorithmic formulation. We present a summary in Table 1 that systematically outlines the key algorithmic steps and corresponding mathematical notations for our two primary contributions: the Omnidirectional Channel-Selective Scanning (OCS) module and the Dual-Branch Consensus Thresholding (DBCT) module. This table is intended to serve as a quick reference, facilitating a deeper understanding of the model’s components and their operational flow.

## 4. Experiments

### 4.1. Dataset

Experiments are conducted on PotholeDataset, a road pothole detection corpus of 13,767 images annotated with one class and a uniform resolution of 640 by 640 pixels. The dataset is divided into 9637 training images, 2754 validation images, and 1376 test images (7:2:1). The dataset link is https://github.com/xingt9227-design/Road-Pothole-Detection-Dataset (accessed on 2 December 2025).

### 4.2. Experimental Settings

We trained and evaluated on a single workstation running Ubuntu 22.04 LTS with an NVIDIA RTX 3090 with 24 GB VRAM, an Intel Core i9 13900K CPU, and 128 GB RAM. The environment used Python 3.10, PyTorch 2.3, CUDA 12.1, cuDNN 8.9, the official YOLOv11 [30] repository, OpenCV 4.8, and Matplotlib 3.7.

The input resolution was fixed at 640 × 640. We trained for 300 epochs with a batch size of 16, enabling mosaic augmentation in the early phase and disabling it after epoch 50. Early stopping patience was 50. The optimizer was AdamW with an initial learning rate of 0.001 and cosine annealing to a 0.01 scale, momentum of 0.937, and weight decay of 0.0005. Data caching was off, training used GPU device 0, and outputs were stored in runs/train with experiment id exp_crack. Configuration files governed dataset partition and label schema to support reproducibility and fair comparison.

In the implementation of the OCS and DBCT modules, all training behaviors, including augmentation strategies, assignment rules, and label smoothing, remain unchanged. OCS and DBCT only operate on intermediate feature maps and do not alter the spatial hierarchy or the supervision pipeline of the network. Specifically, the YOLOv11 training framework remains consistent, with Mosaic and MixUp augmentations, HSV and geometric transformations, task-aligned assignment rules, and label smoothing unchanged in all experiments. This is because OCS and DBCT operations are limited to the feature map processing stage and do not affect the augmentation strategies or assignment rules at the input data level, nor do they interfere with the computation of the loss function. Therefore, despite the introduction of OCS and DBCT modules, the training configuration remains unchanged, ensuring the comparability of experimental results and the stability of the training process.

All parameters introduced by OCS and DBCT are optimized jointly with the backbone under the same AdamW optimizer and follow the same cosine learning rate schedule, without creating additional parameter groups or custom learning rate rules. Directional scanning weights, channel aggregation parameters, and difference enhancement coefficients in OCS all inherit the backbone’s initial learning rate (0.001) and decay behavior. The threshold regressor and gating network within DBCT adopt the same unified schedule as well, ensuring that the entire detector is trained in a consistent and comparable manner.

### 4.3. Main Results

**Figure 7:**
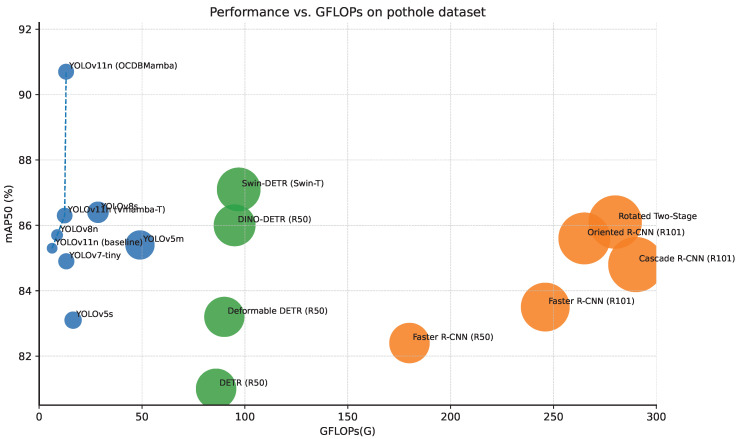
Comparisonof different object detection methods on pothole detection dataset.

As shown in Table 2 and Figure 7, single-stage detectors strike the best balance. With 6.0 M parameters and 13.1 G FLOPs, OCDBMamba attains a precision of 0.905, recall of 0.812, mAP50 of 90.7%, and mAP50–95 of 67.8%, the top scores in all measures.

Over YOLOv11n, mAP50 rises by 5.4 points and mAP50–95 by 5.6%. OCS contributes by establishing row, column, and diagonal long-range context and enforcing cross-directional consistency with channel similarity-based adaptive fusion, which makes responses along thin rims and low-contrast edges more continuous and stabilizes localization under strict thresholds. DBCT adds a feature-level selection map via dual-branch consensus and adaptive thresholds, suppressing activations from shadows and water glare, while boundary consistency regularization preserves true edges and reduces false alarms. Compared with YOLOv8s, we are ahead by 4.3% in mAP50 and 4.1% in mAP50–95, showing that omnidirectional context plus feature-level denoising outperforms pyramid-only upgrades. Against Oriented R CNN, we gain 4.9% in mAP50–95 with roughly one twentieth of the computation, and against Swin DETR we obtain 3.6% in mAP50–95 with about one seventh of the compute, both favorable for real-time deployment. Adding Vmamba T to YOLOv11n improves mAP50–95 from 62.2% to 63.5% but leaves cross-directional cooperation and redundancy suppression largely unresolved. Overall, OCS and DBCT together deliver better accuracy and robustness at comparable complexity.

### 4.4. Ablation Study

**Figure 8:**
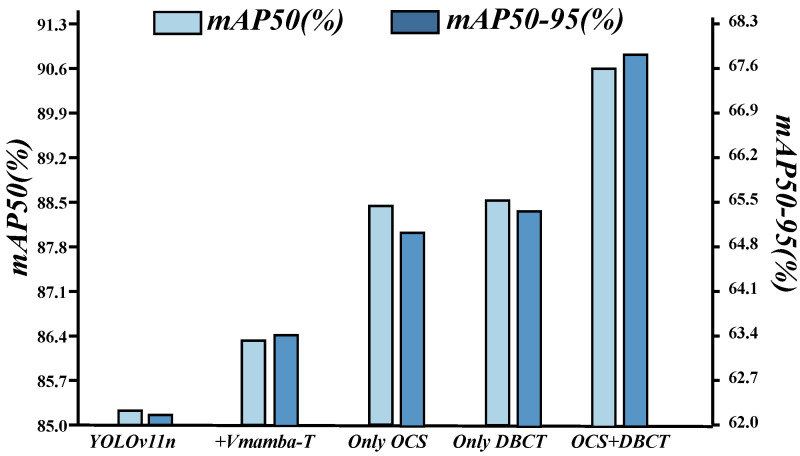
Effect of OCS and DBCT combination.

**Effect of OCS and DBCT combination:** Table 3 and Figure 8 highlight three trends. First, YOLOv11n attains mAP50 85.3% and mAP50–95 62.2%; swapping in Vmamba T nudges these to 86.3% and 63.5%, so longer-range modeling by itself is not sufficient. Second, OCS raises mAP50 to 88.5% and mAP50–95 to 65.0% and improves recall from 0.785 to 0.798 by injecting cross-directional context that smooths weak edges and stabilizes localization. Third, DBCT alone reaches mAP50 88.6% and mAP50–95 65.2% and increases precision from 0.891 to 0.900 by suppressing sporadic activations from shadows and water through a feature-level selection mechanism with adaptive thresholds and boundary consistency. Joint use achieves 90.7% and 67.8% in mAP50 and mAP50–95, a further gain of about 2.1 to 2.8% over single-module use. OCS provides direction-consistent context that grounds DBCT threshold regression, and DBCT filtering lowers fusion conflicts for OCS, keeping long-range responses continuous on narrow rims. Precision and recall rise together under tighter thresholds.

**Figure 9:**
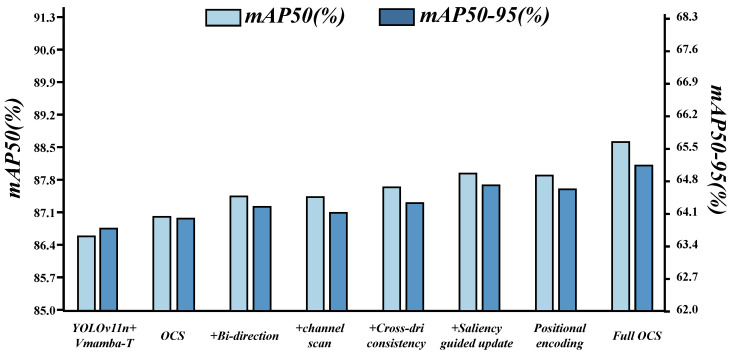
Fine-grained ablation of OCS.

**Fine-grained ablation of OCS:** Table 4 and Figure 9 now evaluate each OCS design choice independently rather than in a cumulative manner.

The first row reports the backbone with Vmamba-T but without OCS, giving 0.894/0.789 in precision/recall and 86.6/63.7 in mAP50/mAP50–95.

Endowing the base OCS variant with single-direction scanning and no regularization already improves performance to 87.0/64.0. When only bidirectional scanning on (h,w) is added on top of the base OCS, mAP50 reaches 87.3 and mAP50–95 reaches 64.2, confirming that symmetric propagation reduces single-direction bias.

With only channel dimension scanning enabled, the detector achieves 87.2/64.1, illustrating that modeling cross-channel similarity helps reconnect semantics in low-contrast regions. Activating only the cross-direction consistency constraint yields 87.4/64.3, indicating that stabilizing directional responses improves fusion quality.

Saliency-guided updates, when used as the sole regularization on top of the base variant, lead to 87.8/64.7 by concentrating updates where information is dense and curbing noise spread at roughly constant cost.

Positional encoding alone gives 87.6/64.5, suggesting that explicit phase position cues help maintain continuity along thin edges and near boundaries.

When all factors are enabled simultaneously in the full OCS configuration, precision/recall reach 0.899/0.798 and mAP50/mAP50–95 reach 88.5/65.0, showing that the individual gains from directional, channel-wise, consistency, saliency, and positional cues are complementary rather than redundant.

**Figure 10:**
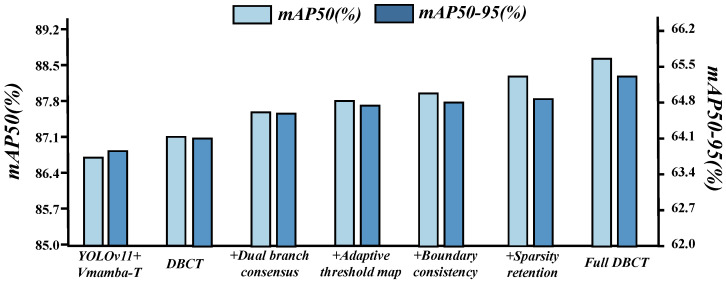
Ablation of DBCT components.

**Ablation of DBCT components:** Table 5 and Figure 10 report a disentangled ablation where each DBCT mechanism is evaluated independently on top of the same base design.

Compared with the backbone without DBCT (86.6/63.7), introducing a single-branch DBCT module already improves performance to 87.1/64.1 by turning noisy activations into a more selective mask.

When only dual-branch consensus is added, precision/recall become 0.896/0.793 and mAP50/mAP50–95 reach 87.6/64.5, showing that multiplicative agreement between branches effectively suppresses reflection- and shadow-induced spikes.

Using only the adaptive threshold map yields 87.8/64.7 by aligning selection strength with local statistics, which stabilizes screening in weak-contrast regions. Boundary consistency, when applied as the sole regularizer, improves the metrics to 87.9/64.8 and preserves true rims after selection, reducing localization drift along pothole edges.

Applying only sparsity retention leads to 88.1/64.9 and slightly higher precision, indicating that global sparsity control encourages compact, discriminative evidence. The full DBCT configuration, which combines consensus, adaptive thresholds, boundary guidance, and sparsity, achieves 0.900/0.800 in precision/recall and 88.6/65.2 in mAP50/mAP50–95, demonstrating that these mechanisms contribute additively rather than merely reparameterizing each other.

**Hyper-parameter sensitivity for OCS and DBCT:** Table 6 presents stable and monotonic gains together with clear optimal zones. In OCS, ϵ=0.5 reaches mAP50 88.5% and mAP50–95 65.0%, beating ϵ=0.3 and ϵ=0.8. When ϵ is too low, the gate becomes rigid and weak-contrast edges fracture; when it is too high, the filter loosens and redundancy grows. A learnable positional encoding amplitude yields 88.5% and 65.0% versus 88.2% and 64.8% without positional encoding, confirming that a learnable phase restores the relative position lost to serialization and improves alignment of thin rims and boundary-adjacent instances. In DBCT, δ=0.5 attains mAP50 88.6% and mAP50–95 65.2%, exceeding δ=0.3 and δ=0.8. Too low a threshold temperature over-hardens selection and prunes true edges; too high a threshold temperature under-selects and introduces texture artifacts. The best sparsity target is ϱ=0.30 with 88.6% and 65.2%; at ϱ=0.25 or ϱ=0.35 the metrics soften, reflecting the detail versus noise trade-off. Defaults sit near the flat optimum, and the consistent increase in mAP50–95 shows better localization at tighter IoU thresholds with minimal tuning effort.

**Training settings:** Performance trends and mechanisms are aligned in Table 7. OCS only with a gating kernel k=5 achieves mAP50 88.5% and mAP50–95 65.0%, superior to k=3 and k=7: the medium receptive field preserves directional coherence and restrains texture, whereas the small one misses cross-directional ties and the large one induces smoothing and aliasing. DBCT only with a hard mask trained by straight-through estimation attains mAP50 88.6% and mAP50–95 65.2%, edging out the soft mask at 88.4% and 65.1%, because the shared threshold aligns discrete inference with a differentiable training path, cleanly pruning redundancy while the boundary consistency constraint protects true rims. In the full pipeline, non-maximum suppression with IoU 0.50 is optimal at mAP50 90.7% and mAP50–95 67.8%. Lowering to 0.45 under-suppresses duplicates and hurts precision, whereas raising to 0.55 over-suppresses and causes merges and misses near adjacent or boundary-adjacent targets. Together, OCS yields continuous cross-directional features for stable candidate ordering and DBCT removes reflection and shadow noise before non-maximum suppression, enabling a better precision–recall trade at mid-IoU and consistently improving localization under stricter thresholds without notable cost.

**Figure 11:**
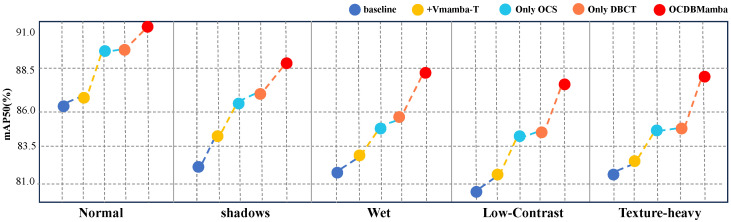
Robustness by scenario subsets (mAP50). The x-axis categories represent different testing environments. Each marker style and color correspond to a specific method (see legend). The exact performance values (y-axis) for each method and scenario (x-axis) are quantitatively presented in Table 7.

**Robustness by scenario subsets:** Table 8 and Figure 11 report consistent gains of OCDBMamba on every subset. Normal scenes improve to 91.2 in mAP50 and 67.9 in mAP50–95 from 86.1 and 62.8. Relative boosts in shadow, water, low contrast, and strong texture are 6.4 and 6.0, 6.5 and 5.9, 6.7 and 5.7, and 6.5 and 5.7 points. These gains are larger and steadier than replacing the backbone alone, showing that memory extension without cross-directional coupling and artifact suppression is insufficient. OCS alone increass low contrast and strong texture to mAP50 84.6% and 85.0% through bidirectional scanning that preserves context on thin and weak edges. DBCT alone increases shadow and water to mAP50 86.3% and 85.6% by turning reflection and dark edge bursts into sparse discriminative evidence via branch consensus and adaptive thresholds. Jointly, OCS stabilizes the statistics used by DBCT threshold regression and DBCT reduces directional conflicts during fusion, yielding the best mAP50 and mAP50–95 on all five subsets with near-baseline latency and compute.

**Overhead Analysis of OCS Components:** Table 9 quantifies the computational and memory characteristics of each OCS sub-module. Directional scanning introduces only 0.42 GFLOPs and 0 MB memory movement because all four spatial scans are implemented through view/reshape-based index remapping without triggering any tensor copying. Channel scanning (Flatten) similarly incurs zero additional memory movement. The channel similarity fusion step in Equations (10) and (11) operates on pooled vectors of dimension *C* = 64, adding only 0.07 GFLOPs and a merge cost below 0.1 ms. The structural difference operator adds 0.11 GFLOPs and a trivial 0.02 MB buffer. Overall, the entire OCS module increases computation by only 0.78 GFLOPs (6.0% of the model) and introduces a total runtime overhead of 2.5% with practically no memory transfer cost. These results confirm that OCS does not suffer from the excessive tensor movement or merging overhead typically associated with multidirectional serialization.

### 4.5. Visualization

**Figure 12:**
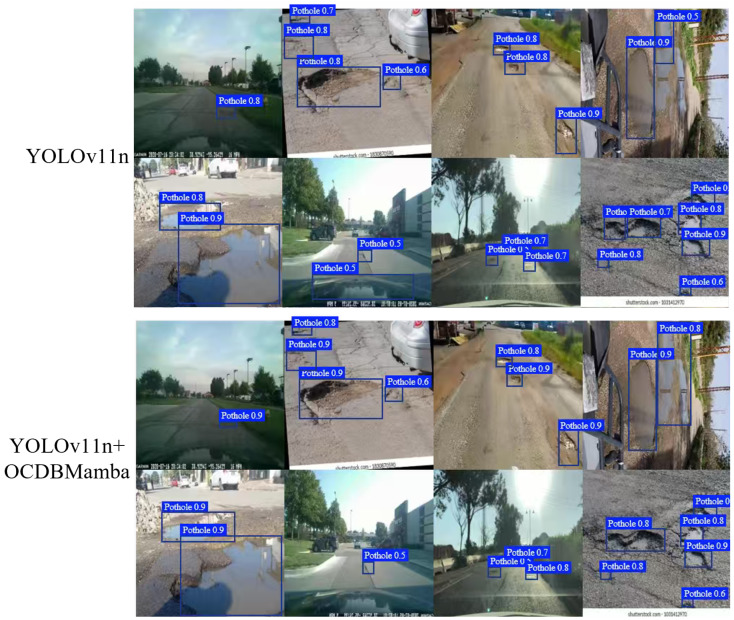
Comparison between baseline and our method.

As shown in Figure 12, under weak illumination the baseline reports a confidence of 0.8 on the first image, whereas our model gives 0.9. In a region with dense fractured texture (second row, fourth column), the baseline splits one damage into two adjacent boxes, while our model merges them into a single coherent box with cleaner boundaries. However, there are still some limitations. For instance, in detecting the large pothole at the bottom of the second image in the second row, our model missed it. Nonetheless, in all other images, our performance was superior to that of the baseline. These gains arise from two components used together: Omnidirectional Channel-Selective Scanning provides cross-directional long-range context with direction consistent fusion, and Dual-Branch Consensus Thresholding performs adaptive feature-level selection with boundary consistency to preserve true rims. Their synergy reduces false alarms and stabilizes localization for small targets and thin boundaries under strong interference and weak contrast, improving practical robustness and deployability.

### 4.6. Model Generalization Testing

**Figure 13:**
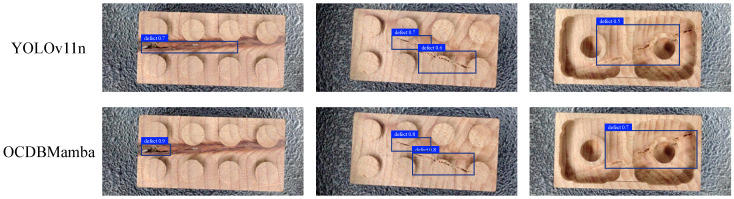
Experimental evaluation of model generalization in wooden workpiece defect detection.

We also annotated 50 images of wood workpiece defects to conduct model generalization experiments on OCDBMamba. As shown in Figure 13, the confidence level of OCDBMamba for workpiece defect detection is higher than that of the baseline YOLOv11 by 0.1 0.2. Thus our model has better generalization than YOLOv11, indicating that our model is not only effective in the road pothole detection task, but also in other anomaly detection tasks, such as abnormal defects in workpieces.

## 5. Conclusions

We present a unified detection framework for road pothole analysis that couples Omnidirectional Channel-Selective Scanning and Dual-Branch Consensus Thresholding under a single end-to-end objective. OCS builds long-range dependencies along row, column, and diagonal paths, using channel-adaptive fusion and cross-directional consistency to strengthen global representation. DBCT forms robust responses via dual-branch consensus, applies adaptive thresholds with sparsity control to suppress redundancy at the feature level, and enforces boundary consistency to preserve true edges, achieving accuracy, robustness, and real-time efficiency at near-linear complexity.

Experiments on subsets covering normal scenes, shadows, wet roads, low contrast, and texture-heavy backgrounds show consistent gains over a YOLOv11 baseline, with stable improvements in both mAP50 and mAP50–95. Ablations evidence complementarity: OCS improves cross-directional context continuity and localization quality, while DBCT suppresses spurious activations from reflections and shadows and stabilizes recall on weak edges. Hyper-parameter sensitivity indicates that defaults lie in a flat optimum, supporting reproducibility and practical deployment.

Despite these encouraging results, our approach has certain limitations. The current framework relies solely on RGB imagery and does not leverage geometric or multimodal sensor inputs such as LiDAR or thermal data, which could further enhance robustness under extreme lighting or severe weather. While the model generalizes well to wooden workpiece defects, its performance on other anomaly types or in cross-domain scenarios with significant appearance shifts remains to be thoroughly validated. The serialization process in OCS, although efficient, may still introduce minor spatial discontinuities at very high resolutions or under extreme perspective distortion. Additionally, the dependency on a fixed input resolution (640 × 640) may limit flexibility in multi-scale or ultra-high-resolution applications. Furthermore, the computational advantages of the method have been validated primarily on a single-class dataset, and their scalability to multi-class detection tasks with more complex label distributions warrants further investigation.

In summary, the framework tightly integrates state space-driven omnidirectional scanning with consistency-driven feature selection, reducing reliance on post-processing and mitigating spatial discontinuities from serialization. With lightweight operators and modest compute, it substantially improves robustness under complex road conditions. Future work includes synergy with three-dimensional geometry and multimodal sensing, domain adaptation across-regions, self-supervised pretraining, and efficient integration with general-purpose vision foundation models.

## Figures and Tables

**Table 1:** Summary of key algorithms and notations in OCDBMamba.

Component	Step	Description	Key Symbols and Equations
Omnidirectional Channel-Selective Scanning (OCS)	1. Input	Unified input feature map representation.	$X\in R^{H\times W\times C}$
	2. Omnidirectional Spatial Scanning	Unfolds the feature map along four directions and captures long-range dependencies via recurrent state updates.	$\begin{array}{cc} S_{\mathrm{spatial}}^{\left(d\right)} & ={\mathrm{Scan}}_{d}\left(X\right)\\ h_{t}^{\left(d\right)} & =f(h_{t-1}^{\left(d\right)},S_{\mathrm{spatial},t}) \end{array}$
	3. Spatial Aggregation	Fuses the scan results from the four directions to form a spatially robust representation invariant to rotation.	$Y_{\mathrm{spatial}}=\frac{1}{4}\sum_{d\in D_{\mathrm{spatial}}}\mathrm{Reshape}\left(h^{\left(d\right)}\right)$
	4. Channel-selective Fusion	Models inter-channel dependencies by computing a channel similarity matrix $M_{ij}$ to adaptively enhance features of relevant channels.	${\hat{Y}}_{\mathrm{ch},i}=\sum_{j}M_{ij}Y_{\mathrm{ch},j}$
	5. Local Structural Difference Enhancement	Uses a spatial difference operator $\Delta_{p}$ to capture and enhance the sharp edges and local contrast of potholes.	$Y_{\mathrm{diff}}=X+\gamma \Delta_{p}$
	6. Final OCS Output	Linearly combines the three representations (spatial, channel, and difference-enhanced) to form the final context-aware feature.	$Y_{\mathrm{OCS}}=\alpha Y_{\mathrm{spatial}}+\beta {\hat{Y}}_{\mathrm{ch}}+\eta Y_{\mathrm{diff}}$
Dual-Branch Consensus Thresholding (DBCT)	1. Input	Feature map from the preceding layer.	*X*
	2. Dual-Branch Processing	Generates two complementary feature branches using different activation functions (Swish, Tanh) to capture diverse feature patterns.	$\begin{array}{cc} E_{1} & =\mathrm{Swish}(X)\\ E_{2} & =\mathrm{Tan}h(X) \end{array}$ $\begin{array}{cc} E_{1} & =\mathrm{Swish}(X)\\ E_{2} & =\mathrm{Tan}h(X) \end{array}$
	3. Adaptive Threshold Map Generation	Regresses a pixel-wise adaptive threshold map *T* from the concatenated features of the two branches.	$T=\sigma (\mathrm{Conv}\left(\mathrm{Concat}\left(E_{1},E_{2}\right)\right))$
	4. Hard-Mask Application with STE	Uses the upsampled threshold map $T_{\mathrm{up}}$ to suppress low-activation regions, while ensuring gradient flow with the straight-through estimator (STE).	${\bar{E}}_{k}=E_{k}⊙1_{E_{k}\geq T_{\mathrm{up}}}$
	5. Consensus-based Fusion	Weights and fuses the thresholded results from both branches using a learned selection weight map *A* to enforce consensus.	$P=a_{1}⊙{\bar{E}}_{1}+a_{2}⊙{\bar{E}}_{2}$
	6. Loss Formulation	Adopts a composite loss function for end-to-end optimization, including the detection loss $L_{\det}$ and the threshold regression loss $L_{\mathrm{thresh}}$.	$L_{\mathrm{DBCT}}=E\left[L_{\det}\right]+\lambda L_{\mathrm{thresh}}$

**Table 2:** Comparison of different object detection methods on pothole detection dataset. Bold indicates best performance in each column.

Detector	Method	Backbone	Params (M)	FLOPs (G)	*P*	*R*	mAP50 (%)	mAP50–95 (%)
One-stage (YOLO Series)	YOLOv5s [31]	CSPDarknet53 [32]	7.2	16.5	0.882	0.762	83.1	60.4
	YOLOv5m [33]	CSPDarknet53	21.2	49.0	0.887	0.775	85.4	62.7
	YOLOv7-tiny [34]	E-ELAN [35]	6.2	13.2	0.885	0.770	84.9	61.8
	YOLOv8n [36]	C2f-FPN [37]	3.2	8.7	0.889	0.780	85.7	62.9
	YOLOv8s [38]	C2f-FPN	11.2	28.6	0.892	0.788	86.4	63.7
	YOLOv11n (baseline) [39]	RepNCSPELAN [40]	2.6	6.3	0.891	0.785	85.3	62.2
	YOLOv11n	Vmamba-T [22]	5.8	12.4	0.894	0.790	86.3	63.5
	YOLOv11n	OCDBMamba (ours)	6.0	13.1	**0.905**	**0.812**	**90.7**	**67.8**
Two-stage Detectors	Faster R-CNN [41]	ResNet50 [42]	41.0	180.0	0.872	0.758	82.4	60.1
	Faster R-CNN	ResNet101 [43]	60.0	246.0	0.880	0.765	83.5	61.2
	Cascade R-CNN [44]	ResNet101	77.0	290.0	0.884	0.770	84.8	62.0
	Oriented R-CNN [45]	ResNet101	68.0	265.0	0.886	0.773	85.6	62.9
	Rotated Two-Stage Detector [46]	Custom (Patch-based)	72.0	280.0	0.890	0.780	86.1	63.3
Transformer-based	DETR [47]	ResNet50	41.0	86.0	0.860	0.742	81.0	59.0
	Deformable DETR [48]	ResNet50	40.5	90.0	0.872	0.760	83.2	61.0
	DINO-DETR [49]	ResNet50	44.0	95.0	0.888	0.775	86.0	63.4
	Swin-DETR [50]	Swin-T [51]	48.0	97.0	0.892	0.781	87.1	64.2

**Table 3:** Effect of OCS and DBCT combination. Bold indicates the optimum value.

Configuration	Precision	Recall	mAP50 (%)	mAP50–95 (%)	Params	FLOPs (G)
YOLOv11n baseline	0.891	0.785	85.3	62.2	2.6	6.3
+Vmamba-T backbone	0.894	0.790	86.3	63.5	5.8	12.4
Only OCS	0.899	0.798	88.5	65.0	6.0	12.6
Only DBCT	0.900	0.800	88.6	65.2	5.8	12.9
OCS + DBCT (OCDBMamba)	**0.905**	**0.812**	**90.7**	**67.8**	6.0	13.1

**Table 4:** Fine-grained ablation of OCS (each factor evaluated independently). Bold indicates the optimum value.

Setting	P	R	mAP50 (%)	mAP50–95 (%)	Params (M)	FLOPs (G)
YOLOv11n + Vmamba-T (w/o OCS)	0.894	0.790	86.3	63.5	5.80	12.40
Base OCS (single-dir, no reg.)	0.895	0.791	86.8	63.8	5.85	12.48
+Bidirection (h, w) only	0.896	0.792	87.1	64.0	5.88	12.52
+Channel scan (c) only	0.896	0.793	87.2	64.1	5.90	12.55
+Cross-dir consistency only	0.897	0.794	87.4	64.3	5.92	12.58
+Saliency-guided update only	0.898	0.796	87.7	64.6	5.95	12.61
+Positional encoding only	0.898	0.796	87.6	64.5	5.94	12.60
Full OCS (all factors enabled)	**0.899**	**0.798**	**88.5**	**65.0**	6.00	12.60

**Table 5:** Ablation of DBCT components (each mechanism evaluated independently). Bold indicates the optimum value.

Setting	P	R	mAP50 (%)	mAP50–95 (%)	Params (M)	FLOPs (G)
YOLOv11n + Vmamba-T (w/o DBCT)	0.894	0.790	86.3	63.5	5.80	12.40
Base DBCT (single-branch)	0.895	0.791	87.1	64.0	5.80	12.55
+Dual-branch consensus only	0.896	0.793	87.6	64.3	5.80	12.70
+Adaptive threshold map only	0.897	0.794	87.8	64.6	5.80	12.78
+Boundary consistency only	0.897	0.795	87.9	64.8	5.80	12.83
+Sparsity retention only	0.898	0.796	88.1	64.9	5.85	12.88
Full DBCT (all mechanisms enabled)	**0.900**	**0.800**	**88.6**	**65.2**	5.80	12.90

**Table 6:** Hyper-parameter sensitivity for OCS and DBCT (single-module runs). Bold = default used in OCDBMamba.

Config	Precision	Recall	mAP50 (%)	mAP50–95 (%)
*OCS (Only OCS enabled)*				
ε=0.3 (saliency temp)	0.897	0.796	88.0	64.7
** ε=0.5 **	**0.899**	**0.798**	**88.5**	**65.0**
ε=0.8	0.898	0.797	88.3	64.9
||A||=||B||=0 (no pos-enc)	0.898	0.797	88.2	64.8
**||A||,||B|| learnable**	**0.899**	**0.798**	**88.5**	**65.0**
*DBCT (Only DBCT enabled)*				
δ=0.3 (thr temp)	0.899	0.798	88.3	65.0
** δ=0.5 **	**0.900**	**0.800**	**88.6**	**65.2**
δ=0.8	0.898	0.798	88.2	64.9
ϱ=0.25	0.899	0.799	88.4	65.1
** ϱ=0.30 **	**0.900**	**0.800**	**88.6**	**65.2**
ϱ=0.35	0.899	0.799	88.4	65.1

**Table 7:** Training settings. OCS uses gating conv kernel *k*; DBCT uses hard-mask at test time.

Setting	Precision	Recall	mAP50 (%)	mAP50–95 (%)
*Only OCS*				
k=3	0.898	0.797	88.3	64.9
** k=5 **	**0.899**	**0.798**	**88.5**	**65.0**
k=7	0.899	0.798	88.4	64.9
*Only DBCT*				
Soft mask (no harden)	0.899	0.799	88.4	65.1
**Hard mask (STE)**	**0.900**	**0.800**	**88.6**	**65.2**
*OCDBMamba (Full)*				
NMS IoU = 0.45	0.904	0.810	90.3	67.5
**NMS IoU = 0.50**	**0.905**	**0.812**	**90.7**	**67.8**
NMS IoU = 0.55	0.904	0.813	90.5	67.7

**Table 8:** Robustness by scenario subsets (mAP50/mAP50–95). Bold indicates the optimum value.

Method	Normal	Shadows	Wet	Low-Contrast	Texture-Heavy
YOLOv11n baseline	86.1/62.8	82.7/60.1	81.9/59.7	80.8/58.9	81.5/59.2
+Vmamba-T	87.1/64.0	83.8/61.0	83.0/60.6	82.2/59.6	82.7/60.1
Only OCS	89.1/65.7	86.0/63.4	85.2/62.9	84.6/62.0	85.0/62.3
Only DBCT	89.2/65.9	86.3/63.7	85.6/63.1	84.7/62.2	85.1/62.5
OCDBMamba (full)	**91.2/67.9**	**89.1/66.1**	**88.4/65.6**	**87.5/64.6**	**88.0/64.9**

**Table 9:** Quantified overhead of OCS components. “MemMove” denotes actual memory movement measured in MB; “MergeCost” refers to the fusion overhead during multidirection integration. Bold indicates the optimum value.

Component	FLOPs	MemMove (MB)	MergeCost	Throughput
Directional Scan (4 dirs)	+0.42 G	**0.00**	negligible	−1.3%
Channel Scan (Flatten)	+0.18 G	**0.00**	none	−0.7%
Channel Similarity Matrix *M*	+0.07 G	**0.00**	<0.1 ms	−0.5%
Difference Operator	+0.11 G	0.02 MB	none	−0.3%
**Total Overhead (OCS)**	**+0.78 G**	**0.02 MB**	<0.2 ms	**−2.5%**

## Data Availability

The PotholeDataset is publicly available from its official repository. No new data were created or analyzed in this study.

## Abbreviations

The following abbreviations are used in this manuscript:

OCDBMamba	Omnidirectional Channel-Selective Scanning and Dual-Branch Consensus Thresholding Mamba
OCS	Omnidirectional Channel-Selective Scanning
DBCT	Dual-Branch Consensus Thresholding
